# Atypical Teratoid/Rhabdoid Tumor of the Sellar Region in an Adult Male: A Case Report

**DOI:** 10.7759/cureus.36599

**Published:** 2023-03-23

**Authors:** Wafa Aldhafeeri, Fehid Habelrih, Lina A Alshehri, Jamal Abdullah, Muhammed M Alkutbi, Syed M Shah

**Affiliations:** 1 Neurological Surgery, Prince Sultan Military Medical City, Riyadh, SAU

**Keywords:** smarcb1, inactivation of smarcb1, sellar lesion, at/rt, atypical teratoid/ rhabdoid tumor

## Abstract

Atypical teratoid/rhabdoid tumor (AT/RT) is a rare, fast-growing, aggressive tumor that is almost exclusively seen in the pediatric population; it has a poor prognosis despite aggressive treatment. Adult cases were thought to be exclusively of women, with a total of 23 cases reported worldwide. We herein report a case of a 35-year-old male who posed a unique clinical and diagnostic challenge. To the best of our knowledge, this is the third case of a male patient with sellar AT/RT in the world.

## Introduction

Atypical teratoid/rhabdoid tumor (AT/RT) was first described in 1985 [1]. It is a rare, fast-growing, aggressive tumor that is mostly exclusively seen in the pediatric population with a poor prognosis despite aggressive management [2]. It constitutes 1-2% of all pediatric central nervous system (CNS) tumors [3]. Nonetheless, there are around 50 cases reported of adults; the average age was 36.69 (SD = 15.19) years with a range from 18 to 59 years [4]. Thirty-nine of these cases are sellar AT/RT in adults to date [5]. AT/RT can occur anywhere in CNS. As with all CNS tumors, clinical presentation varies with tumor location. Interestingly, almost all adult patients with sellar AT/RT were female, which raised the question of it being a sex-related disease.

Herein, we report the fourth case of sellar atypical teratoid/rhabdoid tumor (AT/RT) in a 35-year-old male who presented with a headache and posed a unique clinical and diagnostic challenge. 

## Case presentation

**Figure 1:**
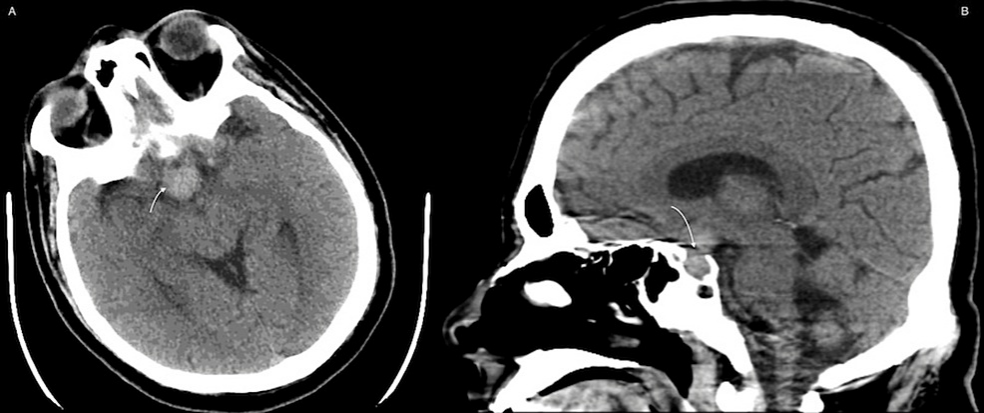
CT scan axial (A) and saggital (B) cuts, showing sellar hyperdensity representing the lesion of interest indicated by the white curved arrows in both cuts

**Figure 2:**
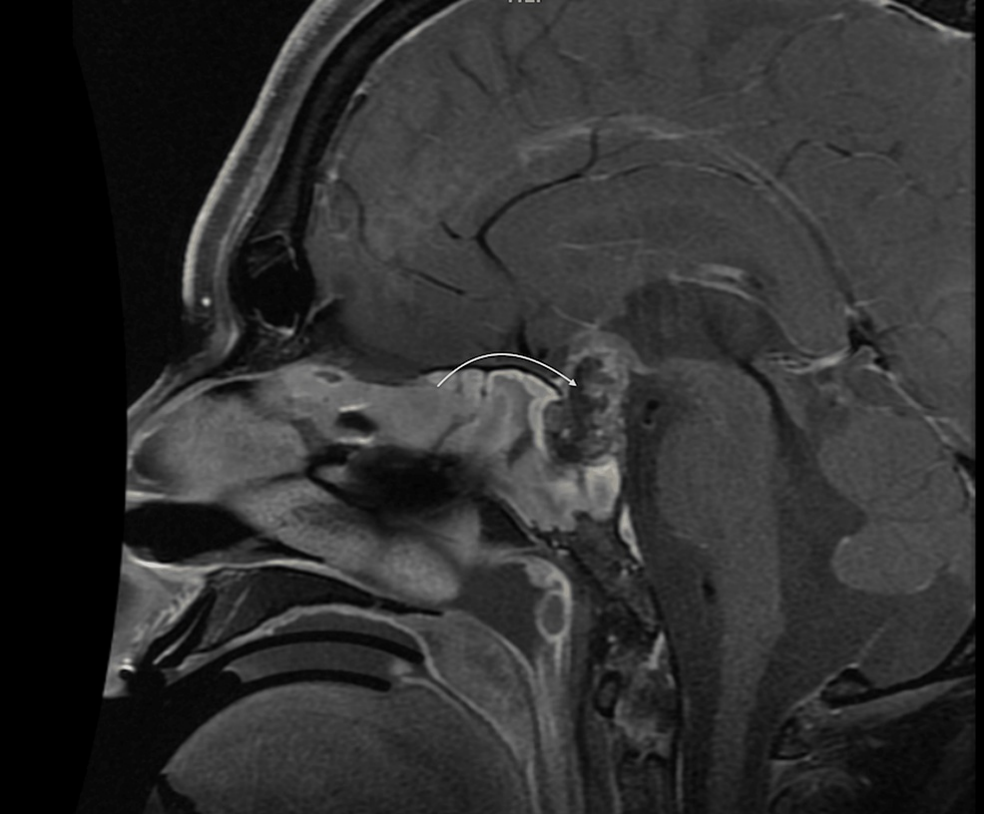
MRI brain saggital cut with contrast showing a sellar lesion with suprasellar extension (curved white arrow)

A 32-year-old male, who is a known case of schizophrenia and obsessive-compulsive disorder, presented to our emergency department as he was complaining of headache, confusion, high-grade fever, and vomiting for 4 days. Also, he had left eye redness and greenish discharge. The patient became unresponsive and dyspneic a few hours after his presentation, so he was intubated and stabilized. Later, the patient developed a generalized tonic-clonic seizure. On examination, his Glasgow Coma Scale (GCS) was 7 and his pupils were equal and reactive. No signs of meningism, clonus, or further abnormal movement were observed, and further neurological assessments, including power and sensation, were limited by the patient's consciousness level. Computed tomography (CT) of the brain (Figure 1) showed a sellar mass with suprasellar extension compressing the chiasmatic structures asymmetrically, right more than left. The lesion invaded the right cavernous sinus and intruded the anterior aspect of the sellar floor. The lesion was considered an incidental finding, and the patient was worked up as a case of meningoencephalitis and started on antibiotics. The management of the mass was deferred temporarily, as the clinical condition of the patient was not suitable. The patient's septic workup, including cerebrospinal fluid (CSF) examination, was negative. The hormonal profile showed panhypopituitarism, thus hormonal replacement was started. On the third day of admission, a diagnosis of submissive pulmonary embolism was made and the patient was started on a therapeutic dose of enoxaparin. MRI was done (Figure 2) to further characterize the seller lesion, the patient was fully covered with antibiotics, anti-seizure medications, and full hormonal replacement therapy; yet, no improvement of the consciousness level was observed. Three weeks into his admission, the patient developed unequal pupils, dilated pupils - left 5 mm and right 3 mm, with ophthalmoplegia (3rd nerve palsy). The CT scan was repeated and showed intra-ventricular hematoma; the therapeutic enoxaparin was stopped and the patient was put on pneumatic compression and an inferior vena naval filter.

**Figure 3:**
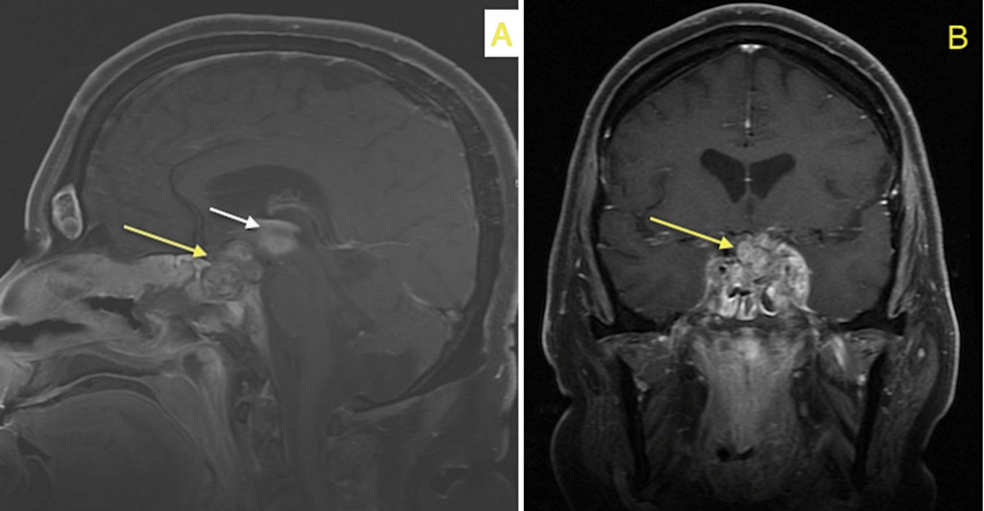
MRI brain with contrast showing slight interval progression of lesion size with heterogeneous texture/enhancement and ill-defined margins in the sagittal (A) and coronal (B) planes (yellow arrows) and the development of subacute intraventricular hemorrhage predominantly involving the third ventricle (white arrow)

The patient had a new MRI (Figure 3), and shortly after that, he underwent emergent trans-sphenoidal endoscopic de-bulking for a sellar/suprasellar lesion. ﻿Postoperatively, the patient’s neurological status remained unchanged.

**Figure 4:**
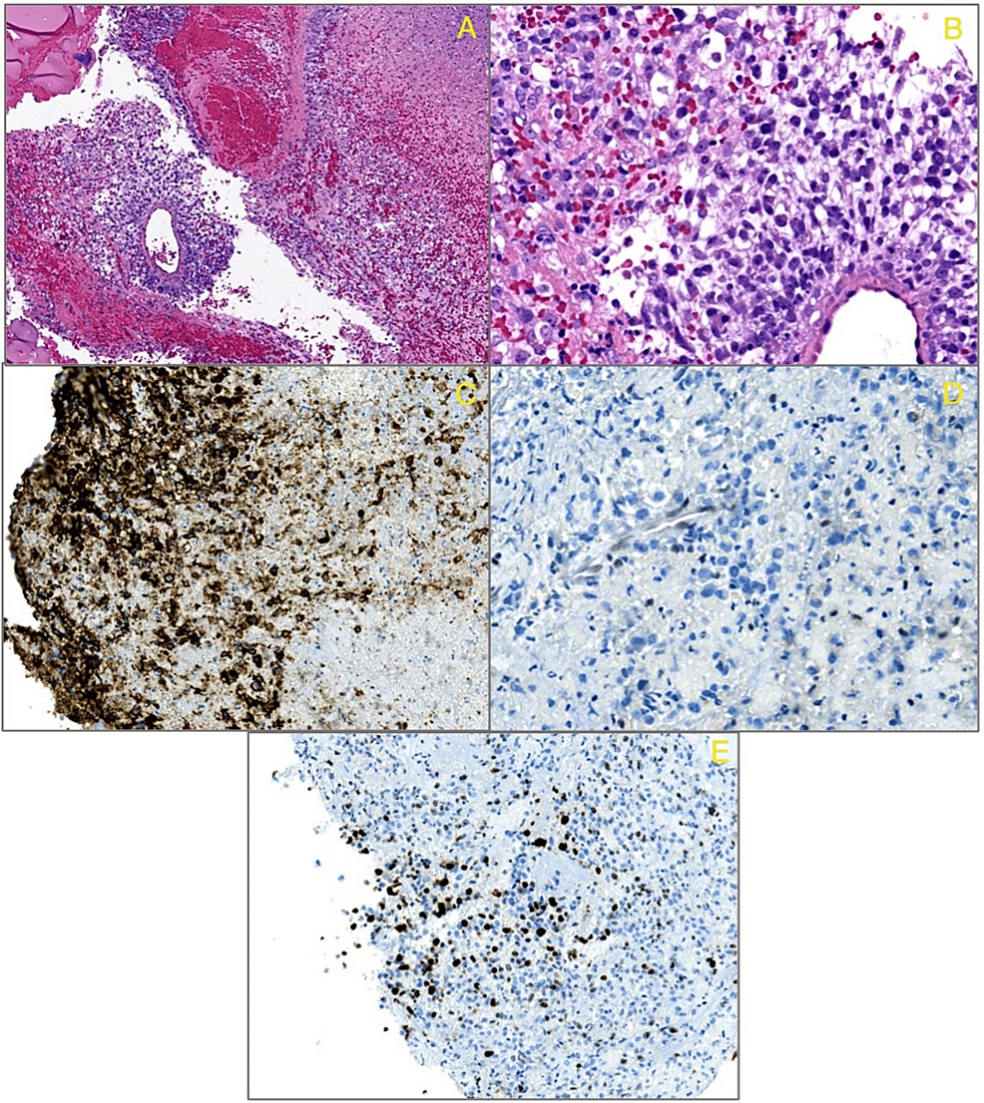
Histopathological examination A: H&E slide shows hemorrhagic and necrotic neoplasm with focal papillary architecture. B: The tumor is composed of cells with round nuclei, prominent nucleoli, and scant cytoplasm. C: Immunohistochemical stain (EMA), positive in the tumor cells. D: Immunohistochemical stain (INI-1), loss of expression in the tumor cells, and retention in the internal positive controls including endothelial cells. E: KI67 proliferation index, focally elevated.

﻿Histopathological examination (Figure 4) showed a highly hemorrhagic and necrotic neoplasm. The residual viable tumor showed focally papillary architecture and was composed of cells with round to ovoid nuclei, prominent nucleoli, and scant cytoplasm. The cells were negative for synaptophysin, prolactin, and transcription factor Pit1. The cells showed a loss of expression of INI-1, ﻿which was consistent with an atypical teratoid/rhabdoid tumor. Immunohistochemical stains showed positivity for epithelial membrane antigen (EMA) and faint patchy positivity for CD56 and had a Ki67 proliferation index focally elevated (5-10%). Pituitary hormones, including prolactin, growth hormone (GH), thyroid-stimulating hormone (TSH), adrenocorticotropic hormone (ACTH), follicle-stimulating hormone (FSH), and luteinizing hormone (LH), were negative.

**Figure 5:**
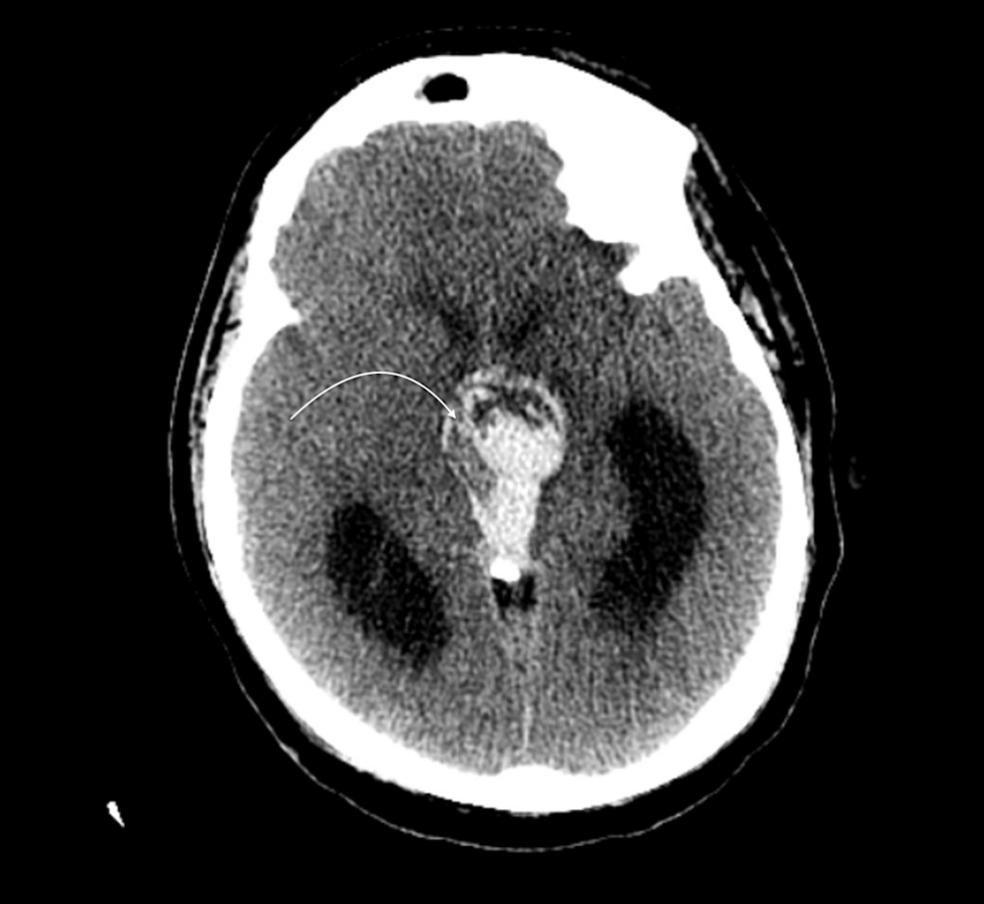
CT scan axial cut showing the third ventricular extension of the hemorrhage (white curved arrow)

The patient was referred to the oncology service for further management. Yet, two weeks after surgery, the patient deteriorated in terms of level of consciousness. His GCS was 3, with absent brainstem reflexes. CT of the brain was done (Figure 5) and showed progressive intra-ventricular hemorrhage and hydrocephalus, with diffuse cerebral edema. An external ventricular drain was inserted but with no improvement clinically. The patient was declared brain dead, and he died three months after the initial presentation.

## Discussion

AT/RT is prototypically an aggressive tumor of infancy, constituting 10% of CNS tumors in this age group [6,7]. AT/RT is defined by the inactivation of SMARCB1 or SMARCA4 in rare cases [7]. The occurrence of AT/RT in adults is extremely rare, with 39 cases reported in the literature to date [5]. Distinguished from pediatric AT/RT, adult-onset AT/RT is typically supra-tentorial, with a slight predilection for mid-line structures [5]. Moreover, many cases reported long-term survival, which may indicate a more favorable outcome than in pediatric cases [8].

To our knowledge, this case is the fourth sellar AT/RT in male patients [5,9]. The underlying mechanism is still unknown, as there was no deviation to a certain age group or significant relation to hormonal profile [10].

Imaging findings are variable and nonspecific. AT/RT has iso-intense to hyperintense T2 signals, which enhance with contrast administration [7]. Histologically, these tumors are heterogenous lesions, composed of bizarre eosinophilic cells with eccentric nuclei, and mixed features of rhabdoid, neuroectodermal, and mesenchymal cells [7].

Loss of INI1 staining ﻿due to SMARCB1 mutations is considered sufficient for diagnosis, which appears to be the most consistent mutation in adult AT/RR [7]. Alternatively, inactivation of BRG1 due to mutation of SMARCA4 is rarely recognized if INI1 expression is intact [11]. Nakata et al. studied the molecular status of the INI1/SMARCB1 gene in a series of adult sellar AT/RT [10]. Compound heterozygous mutations were present in 57% of cases of sellar AT/RT, in contrast to pediatric AT/RT where such mutations are rare [10,11]. In addition, there was a significant difference in their prevalence between sellar AT/RT and conventional AT/RT. On the other hand, homozygous deletion of the INI1 gene was not reported in sellar AT/RT cases despite being a common mutation in typical AT/RT [6]. Those observations may indicate that sellar AT/RT represents a unique variant of AT/RT with different demographical, molecular, and clinical characteristics.

The estimated median overall survival of sellar AT/RT is 30 months, with a one-year survival of 76.7 as compared to 11 to 14 months for conventional AT/RT [9,12]. Achieving gross total resection was a determinant for favorable outcomes in pediatric patients [13]. Yet, there was no survival benefit seen for gross total resection in adult AT/RT [14]. Receiving chemotherapy and radiotherapy was significantly associated with better survival compared with radiotherapy only or no adjuvant therapy at all [14]. Adjuvant therapy protocols varied drastically in the literature, with data mostly being extrapolated from the pediatric literature. Slavc et al. developed an intensive nine-week course of a dose-dense regimen, augmented with intrathecal therapy followed by high-dose chemotherapy and radiotherapy for pediatric AT/RT [8]. This protocol achieved a five-year overall survival rate of 100% and a five-year event-free survival rate of 88.9% in ﻿nine cases of AT/RT with and without a disseminated disease.

Sellar AT/R is extremely rare in an adult male. We report the fourth case in the literature. Sellar AT/RT poses a unique entity that mandates further research and understanding.

## Conclusions

In conclusion, sellar atypical teratoid/rhabdoid tumor (AT/RT) is an extremely rare tumor in males. in the above article, we reported a case of a 35-year-old male who posed a unique clinical and diagnostic challenge. To the best of our knowledge, this is the fourth case of a male patient with sellar AT/RT in the world. More cases are needed to further characterize and deeply understand the pathology. In this case, the rapid and unpredicted deterioration since day one of the patient’s presentation, along with the poor number of cases reported at that time (2019-2020) all contributed to the sub-optimal management. From this case, we conclude that such tumors somehow portend the ability to hemorrhage and thus careful usage of anticoagulants should be carefully studied before initiation. De-bulking of the lesion timing is critical and more cases are needed for comparison purposes, as the clinical course and prognosis of such lesions are guarded.

## References

[1] Almalki MH, Alrogi A, Al-Rabie A, Al-Dandan S, Altwairgi A, Orz Y (2017). Atypical teratoid/rhabdoid tumor of the sellar region in an adult with long survival: case report and review of the literature. J Clin Med Res.

[2] Biegel JA (2006). Molecular genetics of atypical teratoid/rhabdoid tumor. Neurosurg Focus.

[3] Bose KS, Sarma RH (1975). Delineation of the intimate details of the backbone conformation of pyridine nucleotide coenzymes in aqueous solution. Biochem Biophys Res Commun.

[4] Chan V, Marro A, Findlay JM, Schmitt LM, Das S (2018). A systematic review of atypical teratoid rhabdoid tumor in adults. Front Oncol.

[5] Liu F, Fan S, Tang X, Fan S, Zhou L (2020). Adult sellar region atypical teratoid/rhabdoid tumor: a retrospective study and literature review. Front Neurol.

[6] Horn M, Schlote W, Lerch KD, Steudel WI, Harms D, Thomas E (1992). Malignant rhabdoid tumor: primary intracranial manifestation in an adult. Acta Neuropathol.

[7] Isikay I, Hanalioglu S, Basar I, Narin F, Bilginer B (2019). Survival benefit with gross total resection and adjuvant radiotherapy in childhood atypical teratoid/rhabdoid tumors: results of a single-center cohort of 27 cases. Turk Neurosurg.

[8] Louis DN, Ohgaki H, Wiestler OD (2007). The 2007 WHO classification of tumours of the central nervous system. Acta Neuropathol.

[9] Nakata S, Nobusawa S, Hirose T (2017). Sellar atypical teratoid/rhabdoid tumor (AT/RT): a clinicopathologically and genetically distinct variant of AT/RT. Am J Surg Pathol.

[10] Nishikawa A, Ogiwara T, Nagm A (2018). Atypical teratoid/rhabdoid tumor of the sellar region in adult women: is it a sex-related disease?. J Clin Neurosci.

[11] Rickert CH, Paulus W (2001). Epidemiology of central nervous system tumors in childhood and adolescence based on the new WHO classification. Childs Nerv Syst.

[12] Schrey D, Carceller Lechón F, Malietzis G (2016). Multimodal therapy in children and adolescents with newly diagnosed atypical teratoid rhabdoid tumor: individual pooled data analysis and review of the literature. J Neurooncol.

[13] Slavc I, Chocholous M, Leiss U (2014). Atypical teratoid rhabdoid tumor: improved long-term survival with an intensive multimodal therapy and delayed radiotherapy. The Medical University of Vienna Experience 1992-2012. Cancer Med.

[14] Torchia J, Golbourn B, Feng S (2016). Integrated (EPI)-genomic analyses identify subgroup-specific therapeutic targets in CNS rhabdoid tumors. Cancer Cell.

